# Representativeness and external comparability of the InGef research database for epidemiological studies in Germany

**DOI:** 10.1186/s12913-026-14966-5

**Published:** 2026-06-18

**Authors:** M. Alibone, M. Ludwig, D. Obermüller, D. Pawlowska-Phelan, R. Norris, J. Jacob

**Affiliations:** InGef - Institute for Applied Health Research Berlin GmbH, Otto-Ostrowski-Straße 5, 10249 Berlin, Germany

**Keywords:** Real-world evidence, Real-world data, Administrative claims, Healthcare reproducibility of results, Databases, Factual

## Abstract

**Background:**

Claims data from statutory health insurance (SHI) funds are increasingly used in public health research. We evaluated the representativeness and external comparability of the InGef research database (RDB), which contains anonymized claims data from approximately 10 million SHI-insured individuals in Germany, using selected disease-specific indicators.

**Methods:**

A retrospective cohort study combining cross-sectional and longitudinal analyses (2015–2023) was conducted. We assessed (1) follow-up duration, (2) demographic representativeness, and external comparability based on two complementary reference indications (3) incidence and mortality of lung cancer, and (4) prevalence and pharmaceutical treatment of bronchial asthma. All outcomes were compared with external reference data from the German Federal Statistical Office (DESTATIS), the German Centre for Cancer Registry Data (ZfKD), and the SHI Pharmaceutical Index (GKV-AI). Direct age and sex standardization was applied to ensure comparability.

**Results:**

The InGef-RDB showed high concordance with the general German population in terms of age and sex distribution (maximum deviation < 0.6 percentage points). 73% of all insured persons remained continuously observable over 9 years. 6.9% died and 19.5% left the database due to changes in their insurance provider, corresponding to an average annual attrition rate of approximately 2.4%. Overall mortality was slightly lower than national statistics (− 0.3 to − 0.4 percentage points). Between 2016 and 2022, the standardized asthma prevalence rose from 5,938 to 6,602 per 100,000 population, before decreasing to 5,977 per 100,000 in 2023. Monoclonal antibody prescription rates for asthma deviated by < 0.01 percentage points from SHI benchmarks. Lung cancer incidence (2016–2022) averaged 41.8 cases per 100,000 persons, closely matching ZfKD data.

**Conclusions:**

The InGef-RDB demonstrates strong demographic alignment and good external comparability across distinct epidemiologic and health care use scenarios. Based on these evaluations, it represents a valid and reliable real-world data source for population-based epidemiological and health services research, provided that appropriate methodological adjustments are applied.

**Supplementary Information:**

The online version contains supplementary material available at 10.1186/s12913-026-14966-5.

## Background

Real-world evidence (RWE) has gained increasing importance in global health research. RWE studies, based on data from routine clinical practice (real-world data, RWD), offer essential insights into disease burden, treatment patterns, therapy adherence, as well as the safety and long-term effects of medical interventions under real-life conditions [1–3]. In this context, secondary data represents a key foundation for epidemiological and health science analyses. In Germany, major sources of secondary data include mortality and population statistics from the Federal Statistical Office (DESTATIS), population-based health studies (e.g., SOEP, NAKO, KiGGS, SENTINEL surveillance), national and regional registries (e.g., the German Centre for Cancer Registry Data, ZfKD, as well as clinical and epidemiological registries from various federal states), and hospital and healthcare utilization data, such as those derived from the DRG system.

A particularly valuable source of data are the claims data from statutory health insurance (SHI). These data provide a comprehensive view of healthcare delivery, covering most sectors and medical provisions where medical providers treat patients insured under SHI. With approximately 90% of the German population covered by SHI, these datasets enable detailed, practice-oriented analyses of morbidity, mortality, treatment pathways, and healthcare utilization.

However, using SHI claims data for epidemiological research entails both methodological and substantive challenges. Challenges include limited clinical detail, missing data on non-reimbursed services, and difficulties in defining control groups [4]. Since these data are primarily collected for billing purposes, they may contain informational gaps and structural biases. Such limitations raise significant concerns about validity, representativeness, and generalizability of findings. Therefore, careful evaluation of the underlying data source is essential to ensure reliable conclusions in health services research.

The InGef research database (InGef-RDB) is a well-established claims database in Germany, widely used in epidemiological and health service research [5–8]. It consists of anonymized statutory health insurance (SHI) claims data from approximately 10 million individuals covered by around 50 SHI funds (at the time period covered, exclusively company-based health insurance funds (Betriebskrankenkassen) in Germany, representing about 11% of the German population and roughly 13% of all SHI members, thereby providing substantial population coverage for comparative epidemiological analyses. Company-based health insurance funds were historically established by individual employers to insure their workforce and have traditionally covered predominantly employed, working-age populations and their dependents. Today, these funds are generally open to all eligible members of the German SHI system.

Given its growing relevance for population-based research, it is essential to assess how accurately the InGef-RDB reflects the general German population and whether it is suitable for long-term epidemiological analyses that rely on valid adjustment and external comparability. Accordingly, representativeness in this study is conceptualized as the external comparability of key epidemiological measures derived from selected, methodologically distinct reference indications, rather than as an exhaustive assessment of all disease entities represented in the database.

Previous studies have primarily evaluated representativeness using broad distributions of diagnoses and drug classes, thereby providing a comprehensive but less methodologically focused assessment [9, 10]. In contrast, the present study aims to evaluate key methodological dimensions of the database by examining a limited number of well-defined and epidemiologically distinct conditions, allowing for a more nuanced assessment of external comparability across different patterns of disease burden and healthcare utilization.

Asthma and lung cancer were selected as complementary reference indications representing distinct epidemiological and healthcare dimensions. Asthma is a common chronic condition predominantly managed in the outpatient setting, characterized by long disease duration, recurrent healthcare contacts, and the use of both standard and specialized pharmacotherapies. In contrast, lung cancer represents a relatively rare but severe condition with high mortality, substantial inpatient care, and well-established nationwide cancer registry data, enabling robust external comparisons. Together, these conditions allow for the evaluation of whether the InGef-RDB adequately captures variation in disease frequency, severity, care settings, and treatment pathways.

The aim of this study was therefore not to exhaustively assess all diagnostic or therapeutic entities within the database, but to examine whether key epidemiological measures derived from methodological distinct and representative use cases are comparable to external reference data when appropriate methodological adjustments are applied. Specifically, we aimed to (i) describe observation periods and follow-up of insured individuals, (ii) analyze sociodemographic characteristics and assess their representativeness compared with the German general population, (iii) estimate the incidence of lung cancer, and (iv) estimate the prevalence of asthma and related medication use, and to compare these estimates with external reference statistics.

## Methods

### Data source and availability

The InGef RDB contains anonymized data from all federal states in Germany and supports both longitudinal analyses, allowing the tracking of individual patient trajectories over time, and cross-sectional analyses across most healthcare sectors. The database provides a rolling observation window of up to 10 years, with data being continuously updated. Across healthcare sectors, data completeness can generally be assumed only after a reporting delay of up to nine months following the end of the planned study period, mainly driven by delays in outpatient billing. In contrast, sector-specific data may be available and complete considerably earlier, with inpatient and pharmaceutical data often reaching completeness within weeks to a few months.

For the current study (November 2025), we used data from 2015 to 2023 and analyzed sociodemographic variables (age, sex, and place of residence), outpatient and inpatient diagnoses, as well as pharmaceutical prescriptions. Diagnoses followed the International Classification of Diseases, 10th Revision, German Modification (ICD-10-GM) [11]. Pharmaceutical data were classified according to the Anatomical Therapeutic Chemical (ATC) system provided by the AOK Research Institute (WIdO) [12]. A detailed overview of the database structure and available variables is provided in supplemental Table 1.

### Study design

This study was conducted as a retrospective, population-based cohort study using anonymized SHI claims data from the InGef-RDB covering the years 2015 to 2023. The study design combined cross-sectional and longitudinal analyses.

To evaluate follow-up duration, all individuals who were insured on January 1, 2015, or born during that year were included and observed until dropout from the database due to disenrollment, death, or the end of the study period (December 31, 2023). Sociodemographic characteristics were analyzed cross-sectionally for each calendar year between 2015 and 2023, examining annual distributions of age, sex, region of residence, and all-cause mortality.

Annual prevalence and incidence estimates were based on persons continuously observable during the respective calendar year; individuals born or died within the year were included accordingly. Patients with bronchial asthma (ICD-10-GM: J45, J46) were identified using either ≥ 2 confirmed outpatient diagnoses in different quarters within one year (M2Q criterion) or ≥ 1 inpatient main or secondary diagnosis.

Persons with at least one monoclonal antibody (mAb) prescription for asthma therapy were identified by ATC-Codes (ATC: D11AH05, R03DX05, R03DX09, R03DX10, R03DX11) and we analyzed the number of prescriptions to validate pharmaceutical data within the InGef-RDB. Incident lung cancer (ICD-10-GM: C34.-) cases were identified using the M2Q outpatient or ≥ 1 inpatient diagnosis criteria. Additionally, ICD-10-GM code C34.9 was only accepted in conjunction with another specific C34.- code to increase diagnostic specificity. Furthermore, all patients with at least one lung cancer diagnosis in a one-year baseline period were excluded from the study population.

For comparability with external reference statistics, all epidemiologic outcomes were directly standardized by age and sex to appropriate population structures. Demographic characteristics and asthma prevalence were standardized to the German general population based on DESTATIS data [13]. Prevalence estimates of patients with mAb prescriptions were standardized to the SHI population using KM6 statistics and compared to prescription and utilization data from the SHI Pharmaceutical Index (GKV-AI), a national information system that documents drug use and expenditures within the German statutory health insurance (https://arzneimittel.wido.de/PharMaAnalyst). Incidence of lung cancer was age-standardized to the European Standard Population 1976 (ESP 1976) and compared with nationwide cancer registry data from the ZfKD, the German Centre for Cancer Registry Data (https://www.krebsdaten.de), which collects and consolidates population-based cancer information from all federal states. The ESP 1976 was applied to ensure methodological consistency and comparability with the ZfKD, which routinely uses this standard for national cancer statistics.

### Statistical analyses

Individual follow-up time was calculated in days for all individuals insured on January 1, 2015, or born in that year. Descriptive analyses were performed for the total cohort and stratified by reason for dropout (disenrollment, death, or end of study). Summary statistics included mean, standard deviation, median, quartiles, and minimum/maximum values. Comparisons of demographic characteristics and all-cause mortality with DESTATIS reference data were conducted by sex, age group, and region. Differences were quantified as absolute deviations in percentage points between the respective distributions. Estimated lung cancer incidence and asthma prevalence were reported per 100,000 persons. Asthma prevalence was directly standardized by age and sex to the German general population while incidence of lung cancer was direct age standardized to the ESP1976. For the analysis of mAb utilization, age- and sex-standardized frequencies were calculated relative to the SHI population. The total number of treated patients and prescriptions, as well as the proportion of mAb prescriptions among all asthma-related prescriptions, were reported. All analyses were conducted in R (version 4.0.2).

## Results

### Sociodemographic representativeness

On average, 7.8 million insured individuals were observed cross-sectionally on January 1 of each year between 2015 and 2023. Comparison of the InGef-RDB with official population data from DESTATIS for the period 2015–2023 indicated good overall agreement with the national population structure (Table 1).

The InGef-RDB demonstrated strong demographic representativeness with respect to sex, with maximum deviations below ± 0.65 percentage points, decreasing to ± 0.12 percentage points by 2023. In contrast, age distributions revealed systematic deviations. Individuals under 40 years were well-represented, with the largest deviation of + 2.1 percentage points observed for women in 2015. The 40–59 age group was consistently overrepresented (maximum deviation: 5.4 percentage points for women in 2019), while older age groups (≥ 60 years) were underrepresented, particularly women aged 60–79 in 2015 (maximum deviation: −5.6 percentage points). These patterns were consistent across both sexes.

Regional distributions also showed structural differences. Individuals residing in western Germany were consistently overrepresented (up to + 8.8 percentage points). These deviations remained stable throughout the observation period.

**Table 1:** Demographic deviations: InGef-RDB vs. DESTATIS

Year	Variable	Strata	InGef (%)	DESTATIS (%)	Absolut difference (%-points)	InGef (%)	DESTATIS (%)	Absolut difference (%-points)
			Female			Male		
2015	Sex	Total	50.1	50.7	-0.6	49.9	49.3	0.6
	Age	0–19	19.6	17.5	2.1	20.7	19.2	1.5
		20–39	25.3	23.4	1.9	24.9	25.6	-0.7
		40–59	33.6	29.2	4.4	33.9	30.5	3.4
		60–79	17.0	22.6	-5.6	17.1	20.6	-3.5
		80+	4.6	7.3	-2.7	3.3	4.2	-0.8
	Region	West	93.5	84.7	8.8	92.6	84.6	7.9
		East	6.5	15.3	-8.8	7.4	15.4	-7.9
2019	Sex	Total	50.2	50.7	-0.5	49.8	49.3	0.5
	Age	0–19	18.6	17.7	1.0	19.8	19.2	0.6
		20–39	23.9	23.5	0.4	24.6	25.7	-1.1
		40–59	33.4	27.9	5.4	32.8	28.9	3.9
		60–79	18.8	22.6	-3.8	18.5	20.8	-2.3
		80+	5.3	8.3	-3.0	4.2	5.3	-1.0
	Region	West	93.7	84.9	8.8	92.9	84.9	7.9
		East	6.3	15.1	-8.8	7.1	15.1	-7.9
2023	Sex	Total	50.5	50.7	-0.1	49.5	49.3	0.1
	Age	0–19	17.6	18.0	-0.4	18.9	19.7	-0.7
		20–39	22.9	23.3	-0.4	24.3	25.7	-1.4
		40–59	31.9	26.5	5.4	30.7	27.1	3.5
		60–79	21.8	23.4	-1.7	21.3	21.7	-0.4
		80+	5.9	8.7	-2.9	4.7	5.7	-1.0
	Region	West	93.6	85.1	8.5	92.8	85.1	7.6
		East	6.4	14.9	-8.5	7.2	14.9	-7.6

### Longitudinal observability

**Fig. 1:**
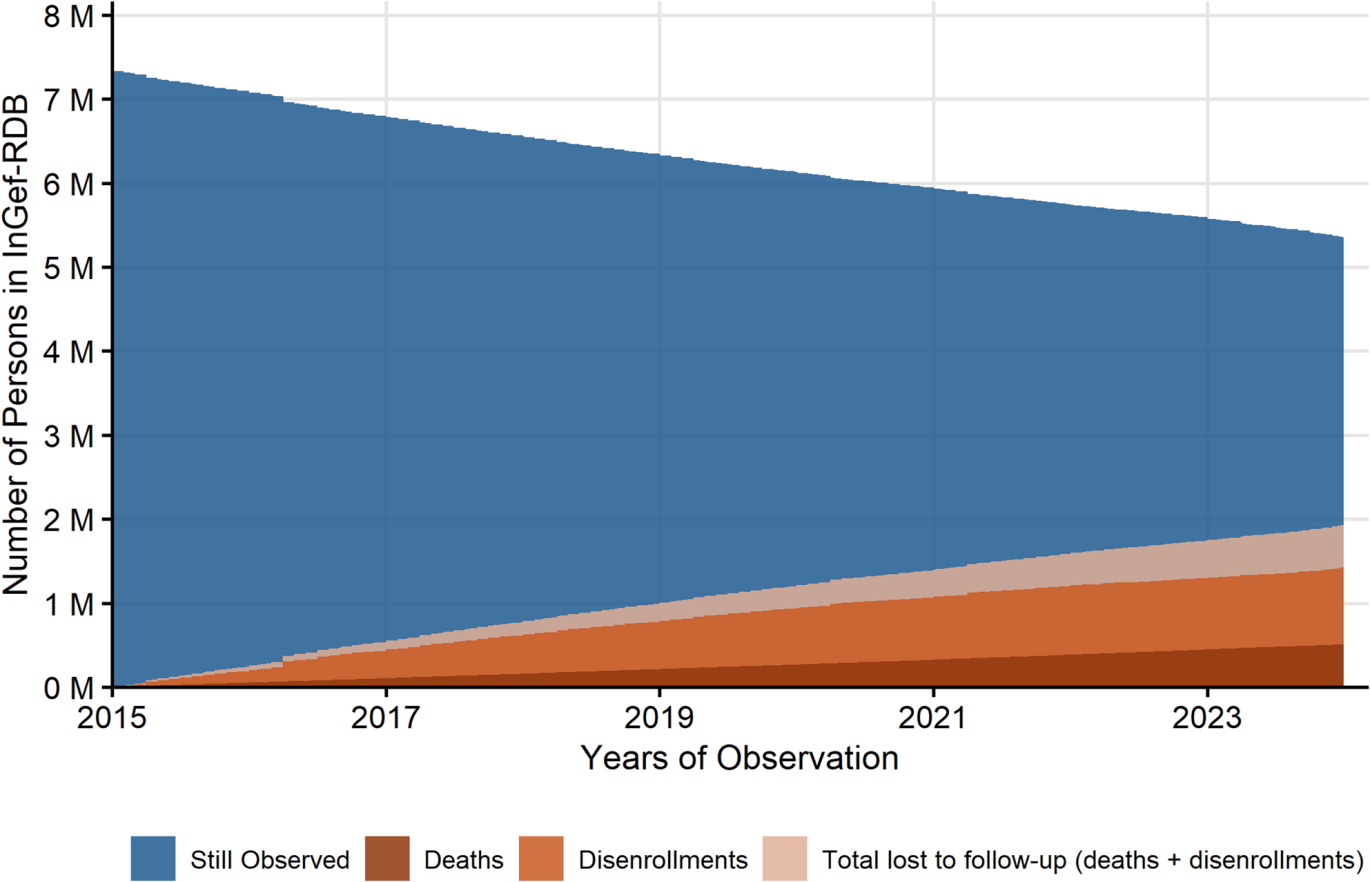
Lost to follow up because of death and disenrollments within the InGef-RDB

Individuals included in the InGef-RDB in 2015 can be followed continuously for an average time of 7.7 years (± 2.5 years). Of these (*n* = 7,335,831), approximately 73% (*n* = 5,399,320) remained continuously insured and were observable without interruption until the end of the study period on December 31, 2023 (Fig. 1). Despite expected dropouts due to death or changes in insurance provider, a substantial proportion of the original cohort was retained over the nine-years.

Among those who did not remain fully observable, 6.9% (*n* = 509,005) died, with a mean follow-up of 4.7 years (± 2.6 years). An additional 19.5% (*n* = 1,427,506) exited the database because of switching to another SHI fund, corresponding to an average annual attrition rate of approximately 2.4%. The mean observation period for individuals who changed insurance provider was 4.1 years (± 2.6 years) between 2015 and December 31, 2023.

### Comparison of the InGef-RDB with DESTATIS mortality statistics

To assess the external comparability of all-cause mortality in the InGef-RDB, annual all-cause mortality from 2015 to 2023 was compared with official DESTATIS statistics (Supplemental Table 2). Overall mortality in the InGef-RDB was slightly lower than in the national statistics, with differences ranging from − 0.4 percentage points in 2015 to − 0.3 percentage points in 2023. Sex-specific analyses indicated fewer recorded female deaths in the InGef-RDB, leading to a systematic overrepresentation of deceased men, with absolute deviations of up to ± 6.1 percentage points. These differences remained stable throughout the observation period. Age-specific analyses showed the largest deviations in the 80 + age group, where women were overrepresented (+ 5.8 percentage points in 2023). Deviations in other age groups were smaller and largely consistent over time.

### Asthma prevalence in the InGef-RDB

**Fig. 2:**
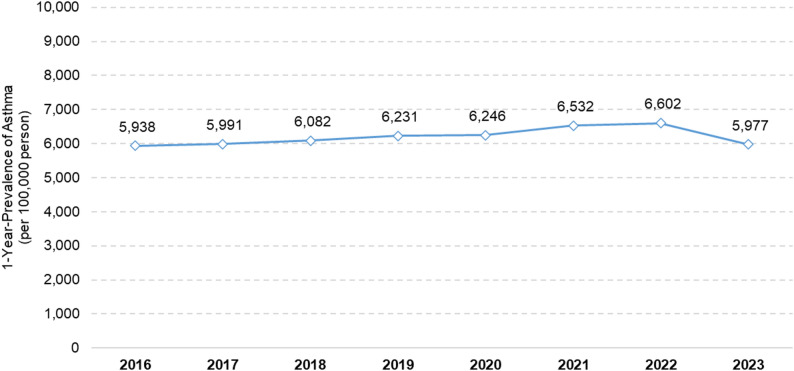
One-year-prevalence of asthma, with age and sex standardized extrapolation to the German population

At the beginning of the observation period in 2016, the estimated age-standardized asthma prevalence for Germany (Fig. 2) was 5,938 cases per 100,000 persons (95% CI: 5.932,5–5.942,7). Prevalence increased steadily to 6,602 per 100,000 persons (95% CI: 6.596,5–6.607,1) by 2022, followed by a notable decline in 2023 to 5,977 cases per 100,000 persons (95% CI: 5,971.5–5,981.6).

### Comparison of mAb-prescriptions for asthma therapy

After extrapolation to the total SHI-insured population (~ 74 million), about 0.09% (*n* = 69,963) of all insured persons received at least one mAb prescription for asthma in 2023. These individuals accounted for 253,580 prescriptions, representing 0.04% of all prescriptions in the extrapolated SHI population (Table 2). Comparison with GKV-AI data showed nearly identical proportions, with a deviation of -0.01 percentage points (absolute difference: -45,920 prescriptions). Dupilumab was the most frequently prescribed mAb, followed by omalizumab.

**Table 2:** Total mAb-prescriptions in 2023 (InGef-RDB extrapolated vs. GKV-AI data)

	InGef-RDB	GKV-AI		
	Sum (%) prescriptions extrapolated to SHI	Sum (%) prescriptions	Absolute difference (%)	Absolute difference (*N*)
**Total**	696,648,860 (100%)	737,302,900 (100%)		
Prescriptions of a monoclonal antibody	253,580 (0.04%)	299,500 (0.04%)	< 0.01	− 45,920
Dupilumab (D11AH05)	139,299 (0.02%)	153,100 (0.02%)	< 0.01	− 13,801
Omalizumab (R03DX05)	51,616 (0.01%)	65,600 (0.01%)	< 0.01	− 13,984
Mepolizumab (R03DX09)	28,754 (0%)	32,300 (0%)	< 0.01	− 3,546
Benralizumab (R03DX10)	18,252 (0%)	27,700 (0%)	< 0.01	− 9,448
Tezepelumab (R03DX11)	15,659 (0%)	20,800 (0%)	< 0.01	− 5,141

Scientific Institute of the AOK (WIdO): Database query on prescription frequencies based on a complete survey of outpatient pharmaceutical prescription data of all statutory health-insured persons, collected pursuant to § 300 SGB V. https://arzneimittel.wido.de/PharMaAnalyst/, Last update: 07 October 2024, Access date: 16 June 2025.

### Comparison of lung cancer incidence between InGef and ZfKD data

**Fig. 3:**
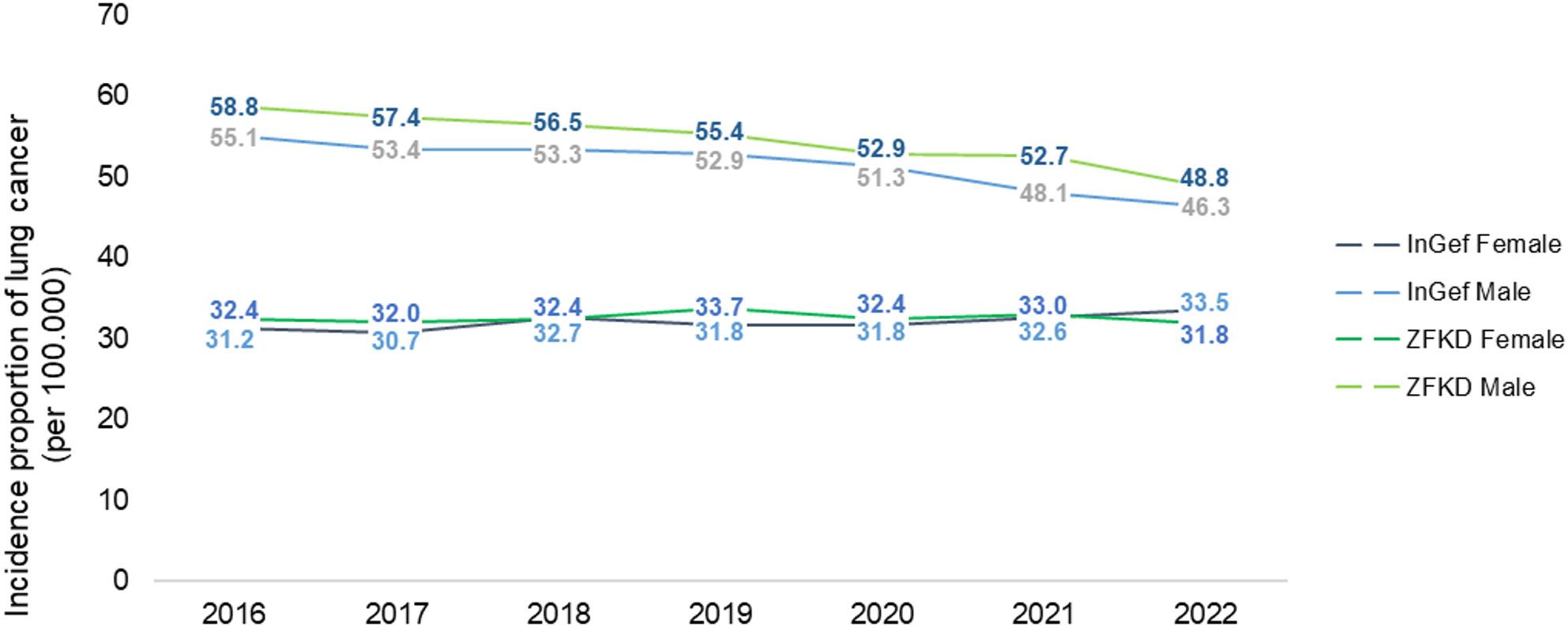
Gender specific incidence of lung cancer age standardized to the ESP 1976 for comparison with ZfKD^1^

In 2016, the incidence of lung cancer in the InGef-RDB was 57.7 cases per 100,000 persons, increasing to 60.2 per 100,000 in 2022 and reaching 66.1 per 100,000 in 2023 (Supplemental Table 3). Sex-specific, age-standardized incidence proportions showed broad agreement with ZfKD cancer registry data (Fig. 3). Among men, standardized incidence proportions in the InGef-RDB were slightly lower than in the ZfKD, with differences ranging from − 1.6 cases per 100,000 in 2020 to − 4.6 in 2021. Deviations among women were smaller, ranging from − 1.9 cases in 2019 to + 1.7 cases per 100,000 in 2022.

## Discussion

This study demonstrates that the InGef-RDB serves as a reliable and robust data source for population-based epidemiological and health services research in Germany when assessed using selected, methodologically distinct reference indications. Comparative analyses of demographic parameters, mortality, disease burden, and medication support the external comparability of key epidemiological measures and the representativeness of the database.

The sex distribution in the InGef-RDB closely aligns with the German population across the entire observation period. Systematic deviations were observed in certain age groups and geographic regions, including a slight overrepresentation of middle-aged adults and insured persons from western federal states. These patterns likely reflect the population structure of the contributing company and guild health insurance funds, whose membership predominantly consists of employed individuals. While these deviations may limit the generalizability for research questions focusing on older adults or regional disparities, such biases can typically be addressed through established methodological adjustments (e.g., direct standardization or stratified analyses). Therefore, demographic characteristics of the insured population should be considered when designing and interpreting studies based on the InGef-RDB.

Longitudinal analyses require stable follow-up to reliably capture clinical outcomes and late events. The InGef-RDB enables continuous observation over up to ten years, supporting long-term analyses. Among individuals enrolled in 2015, approximately 73% remained observable through 2023. Attrition was primarily attributable to:

1. Death: Overall mortality was slightly lower in the InGef-RDB compared with official statistics, consistent with the generally healthier profile of a predominantly employed insured population. Sex- and age-specific deviations indicate the need for appropriate adjustment when estimating mortality outcomes intended to reflect the national population.
2. Approximately 2.4% of insured persons left the database annually due to changes in insurance provider or health fund membership.

Overall, retention patterns indicate high data continuity, confirming the suitability of InGef-RDB for long-term epidemiological research.

Asthma prevalence estimates (5.9–6.6% between 2016 and 2022) fall within the range of previously reported prevalence values and reflect only those patients with documented healthcare utilization [14–18]. Therefore, individuals with mild or well-controlled asthma who did not seek medical care may be underestimated. Prevalence increased steadily until 2022, with pandemic-related effects visible as a temporary stagnation in diagnostic activity during the first COVID-19 year. Estimates from the German Health Atlas (WIdO) show lower prevalence levels, which is expected given its focus on pharmacologically treated asthma and the application of a more restrictive case definition [19]. Differences in temporal trends, particularly during the later pandemic years, likely reflect variations in case definitions and treatment-based validation rather than true epidemiologic divergence. Within the InGef-RDB, the decline in documented asthma prevalence in 2023 may reflect changes in healthcare-seeking behavior, diagnostic coding practices, or treatment strategies following the COVID-19 pandemic.

To assess pharmaceutical care, prescriptions of mAb for severe asthma were analyzed. The proportion of treated patients in 2023 (0.04%) showed excellent alignment with data from the SHI Pharmaceutical Index, supporting strong external comparability for comparative analyses. Minor absolute differences may reflect structural differences in the insured population in the InGef-RDB, including potentially lower morbidity levels, as indicated by slightly lower proportions of patients receiving mAb therapy. Standardized weighting procedures are recommended where precise prevalence estimates of rare or severity-specific conditions are required.

Lung cancer was selected as a clinically relevant condition to evaluate the InGef-RDB’s capacity to estimate key oncological indicators. After age standardization to the ESP 1976, incidence estimates showed close agreement with ZfKD cancer registry data, with only minimal deviations. Moreover, the consistency of relative patterns across sex and age groups demonstrates strong internal validity, ensuring reliability for analyses of risk factors and treatment outcomes. Although clinical details such as tumor stage and histology is lacking due to the billing focus of claims data, the concordant incidence patterns and survival outcomes support the reliability of the InGef-RDB for monitoring disease epidemiology and outcomes in oncology research when using validated case definitions and appropriate methodological safeguards.

The close agreement of diagnosis and medication-based epidemiologic estimates underscores the suitability of the InGef-RDB for pharmacoepidemiological research when appropriate case definitions and methodological adjustments are applied.

Taken together, the analyses across demographic characteristics, longitudinal observability, and two methodologically distinct reference indications demonstrate that the InGef-RDB provides robust and externally comparable epidemiologic measures across different patterns of disease burden and healthcare utilization. On this basis, the database represents a suitable and reliable data source for population-based epidemiological and health services research in Germany.

### Limitations

Several limitations inherent to the InGef-RDB should be considered. First, the database is based on SHI claims and was not collected for research purposes. Hence clinical information is limited and the relevant variables such as tumor stage, laboratory parameters, or lifestyle factors are not available. Second, the database does not allow a detailed assessment of in-hospital medication use, as pharmaceuticals administered during inpatient stays are generally reimbursed via diagnosis-related groups (DRGs) and are not separately recorded at the individual drug level. However, information on certain inpatient drug treatment is available through procedure codes (OPS).

Third, the demographic structure of the database shows minor systematic deviations compared to the German population, particularly a slight overrepresentation of middle-aged adults and an underrepresentation of older age groups, as well as regional imbalances between eastern and western federal states. In addition, comparisons with national population statistics include privately insured individuals, who account for approximately 10% of the German population. Given the structural differences between statutory and private health insurance populations, some deviations in absolute epidemiologic estimates are expected; however, their impact on age- and sex-standardized population-level estimates is likely limited.

Fourth, attrition due to insurance changes and mortality, while generally moderate, may affect estimates of long-term outcomes if not properly accounted for. Finally, the identification of cases and outcomes relies on administrative coding, which can lead to misclassification, especially for conditions with less stringent coding requirements. However, for most epidemiological studies focusing on relative comparisons or intervention effects, internal validity is more critical than representativeness, and the observed differences are unlikely to bias such analyses.

## Conclusions

The InGef-RDB provides a valid and robust foundation for population-based epidemiological and health services research in Germany. The large, longitudinal database enables valid estimates of disease burden and treatment patterns, with high concordance to national reference data. When appropriate methodological adjustments are applied, the InGef-RDB supports both descriptive analyses and comparative effectiveness studies, making it a valuable foundation for real-world evidence generation.

## Supplementary Information

Below is the link to the electronic supplementary material.

Supplementary Material 1

## Data Availability

The data used in this study cannot be made publicly available in the manuscript, the additional files, or in a public repository due to German data protection laws (Bundesdatenschutzgesetz) and legal reasons. In Germany, the utilization of health insurance data for scientific research is regulated by the Code of Social Law. Researchers must obtain approval from the health insurance providers as well as their responsible authorities. As this approval is given only for a specific research question for a specific time and for a specific group of researchers, data cannot be made publicly available. To facilitate the replication of results, anonymized data used for this study are stored on a secure drive at the Institute for Applied Health Research Berlin GmbH (InGef). Access to the raw data used in this study can only be provided to external parties under the conditions of a cooperation contract and can be accessed upon request, after written approval (info@ingef.de), if required.

## Footnotes

1 ZfKD - Center for Cancer Registry Data at the Robert Koch Institute: Database query with estimates of incidence, prevalence, and survival of cancer in Germany based on epidemiological state cancer registry data. Mortality data provided by the Federal Statistical Office. www.Krebsdaten.de, last update: 05.09.2024, access date: 01.04.2025

## Abbreviations

ATC: Anatomical Therapeutic Chemical Classification System
CI: Confidence Interval
COVID-19: Coronavirus Disease 2019
DESTATIS: German Federal Statistical Office
DRG: Diagnosis Related Groups
ESP 1976: European Standard Population 1976
GKV-AI: Statutory Health Insurance Pharmaceutical Index
ICD-10-GM: International Classification of Diseases, 10th Revision, German Modification
InGef-RDB: InGef Research Database
KiGGS: German Health Interview and Examination Survey for Children and Adolescents
KM6: Statutory Health Insurance KM6 statistics
M2Q: Two-Quarter Criterion
mAb: Monoclonal Antibodies
NAKO: German National Cohort
RWD: Real-World Data
SD: Standard Deviation
SHI: Statutory Health Insurance
SOEP: Socio-Economic Panel
WidO: Wissenschaftliches Institut der AOK
ZfKD: German Centre for Cancer Registry Data
