# Supplementary material for: Representativeness and external comparability of the InGef research database for epidemiological studies in Germany
Source: BMC Health Serv Res. 2026 Jun 18;26:853. doi: 10.1186/s12913-026-14966-5 (PMC13282847; doi:10.1186/s12913-026-14966-5)
Supplement: Supplementary file 1 — Supplementary Material 1 [file 12913_2026_14966_MOESM1_ESM.docx]

#### **Supplemental Table 1** Overview of the Variables in the InGef-RDB

| Sector | Area | Content / Description |
| --- | --- | --- |
| Individual Characteristics | Sociodemographic | *Demographic characteristics:*   - Sex - Date of birth (coded by quarter) - Date of death - Nationality (BEV code, DESTATIS)   *Regional characteristics:*   - Place of residence (municipality key – KGS)  Abroad (persons registered abroad)   *Socio-economic characteristics:*   - Person group (e.g. employed persons, students, rehabilitation patients, pensioners, recipients of unemployment benefits ALG I/ALG II, unemployed persons (SGB III), welfare/asylum) - Occupational key (according to KldB 10, including occupational and educational variables) |
|  | Social Insurance Status | *Insurance characteristics:*   - Start and end of insurance period - Insurance status (e.g. retired, family insured) - Co-payment status (none, partial, full exemption) - Participation in special care models (e.g. DMPs, HzV) |
|  | Care Status | *Need for long-term care:*   - Care level (level 1–5) - Date of determination |
| Outpatient Care | Doctors & Medical Care Centres (MVZ) | *Service providers:*   - Doctor’s practice ID (BSNR) - Medical specialty (physician group code from KBV, based on LANR)   *Service utilisation:*   - Medical diagnoses (ICD-10-GM), quarterly  Treatments received (EBM, GOP with exact date) - Outpatient procedures and surgeries (OPS), quarterly - Costs per treatment case   *Special Providers:* Outpatient specialist medical care (ASV), for statutorily insured individuals with rare or complex diseases |
|  | Dentists | *Service providers:*   - Dentist's specialty (dentist ID from KZV, based on ZANR)   *Service utilisation:*   - Treatments and procedures (GOÄ) - Service date - Type of service - Treatment costs (KCH points, IP points and case value) |
|  | Pharmaceuticals | *Service providers:*   - Pharmacy ID (pseudonym) - Pharmacy type (e.g. public pharmacy, hospital pharmacy)   *Service utilisation:*   - Pharmaceutical central number (PZN) - Active ingredients (ATC classification with defined daily doses – DDD) - Quantity dispensed - Prescription and dispensing date - Specialty of the prescribing doctor - Costs of dispensed medication from the health insurer’s perspective (excluding individual discounts between insurers and pharmaceutical companies) |
| Inpatient Care | Hospitals | *Service providers:*   - Hospital ID (pseudonym)   *Service utilisation:*   - Main and secondary diagnoses (ICD-10-GM) - OPS codes (treatments, surgeries) - Admission/discharge date - Reason for admission/discharge (e.g. death in hospital, discharge from hospital) - DRG codes - Costs per case   *Special Providers:* Special care areas (rare diseases with ALPHA_ID_SE and Orpha code) |
| Other forms of Care | Incapacity for work (AU) | *Service providers:*   - Specialty of the certifying physicians   *Service utilisation:*   - Diagnosis of incapacity for work (ICD-10-GM) - Duration of incapacity (start and end) - Sickness benefit claim (KG) - Duration of benefit payment - Amount of sickness benefit |
|  | Remedial and assistive devices | *Service providers:*  *-* Type of provider (e.g. physiotherapy, occupational therapy, medical supply store)  *Service utilisation:*   - Type of service provided - Frequency of treatment - Start and end of utilisation - Costs of remedial and assistive devices |

#### **Supplemental Table 2** Deviation of mortality between InGef-RDB and DESTATIS

| **Year** | **Age Group** | **InGef (%)** | **DESTATIS (%)** | **Absolut difference**  **(%-points)** | **InGef (%)** | **DESTATIS (%)** | **Absolut difference**  **(%-points)** |
| --- | --- | --- | --- | --- | --- | --- | --- |
| **2015** | **Total** | **Total** |  |  |  |  |  |
| **2015** | Total | 0.75 | 1.13 | -0.37 |  |  |  |
| **2019** | Total | 0.78 | 1.13 | -0.35 |  |  |  |
| **2023** | Total | 0.88 | 1.21 | -0.34 |  |  |  |
|  |  | **Female** |  |  | **Male** |  |  |
| **2015** | **Total** | 45.31 | 51.41 | -6.10 | 54.69 | 48.59 | 6.10 |
| **2015** | 0-19 | 0.52 | 0.40 | 0.12 | 0.62 | 0.56 | 0.06 |
| **2015** | 20-39 | 1.02 | 0.66 | 0.36 | 1.78 | 1.53 | 0.26 |
| **2015** | 40-59 | 8.64 | 5.77 | 2.88 | 12.61 | 11.39 | 1.22 |
| **2015** | 60-79 | 29.29 | 27.68 | 1.62 | 43.42 | 44.62 | -1.20 |
| **2015** | 80+ | 60.52 | 65.49 | -4.97 | 41.58 | 41.91 | -0.33 |
| **2019** | **Total** | 44.87 | 50.41 | -5.54 | 55.13 | 49.59 | 5.54 |
| **2019** | 0-19 | 0.47 | 0.38 | 0.08 | 0.53 | 0.56 | -0.03 |
| **2019** | 20-39 | 0.91 | 0.67 | 0.24 | 1.47 | 1.39 | 0.08 |
| **2019** | 40-59 | 8.06 | 5.43 | 2.62 | 10.90 | 10.05 | 0.85 |
| **2019** | 60-79 | 28.40 | 26.49 | 1.92 | 40.58 | 41.21 | -0.63 |
| **2019** | 80+ | 62.16 | 67.03 | -4.87 | 46.53 | 46.80 | -0.27 |
| **2023** | **Total** | 45.10 | 49.99 | -4.89 | 54.90 | 50.01 | 4.89 |
| **2023** | 0-19 | 0.24 | 0.36 | -0.12 | 0.38 | 0.49 | -0.11 |
| **2023** | 20-39 | 0.81 | 0.61 | 0.19 | 1.25 | 1.29 | -0.04 |
| **2023** | 40-59 | 6.66 | 4.69 | 1.97 | 9.59 | 8.66 | 0.93 |
| **2023** | 60-79 | 29.21 | 25.45 | 3.75 | 39.65 | 38.60 | 1.05 |
| **2023** | 80+ | 63.08 | 68.88 | -5.80 | 49.12 | 50.95 | -1.82 |

#### **Supplemental Table 3** Incidence of lung cancer in the InGef-RDB

| InGef | N  patients | Insured persons InGef | Incidence  per 100.000 | 95% CI  lower bound | 95% CI  upper bound |
| --- | --- | --- | --- | --- | --- |
| 2016 | 3,868 | 6,708,711 | 57.66 | 55.87 | 59.50 |
| 2017 | 3,869 | 6,749,314 | 57.32 | 55.55 | 59.16 |
| 2018 | 4,094 | 6,875,128 | 59.55 | 57.75 | 61.40 |
| 2019 | 4,171 | 6,905,946 | 60.40 | 58.59 | 62.26 |
| 2020 | 4,143 | 6,880,513 | 60.21 | 58.41 | 62.07 |
| 2021 | 3,939 | 6,629,647 | 59.41 | 57.59 | 61.30 |
| 2022 | 4,068 | 6,714,951 | 60.58 | 58.75 | 62.47 |
| 2023 | 4,594 | 6,952,621 | 66.08 | 64.19 | 68.01 |
